# A Theranostic Approach to Imaging and Treating Melanoma with ^203^Pb/^212^Pb-Labeled Antibody Targeting Melanin

**DOI:** 10.3390/cancers15153856

**Published:** 2023-07-29

**Authors:** Rubin Jiao, Kevin J. H. Allen, Mackenzie E. Malo, Orhan Yilmaz, John Wilson, Bryce J. B. Nelson, Frank Wuest, Ekaterina Dadachova

**Affiliations:** 1 College of Pharmacy and Nutrition, University of Saskatchewan, Saskatoon, SK S7N 5E5, Canada; ruj501@mail.usask.ca (R.J.); kja782@mail.usask.ca (K.J.H.A.); mem510@mail.usask.ca (M.E.M.); orhan.yilmaz@usask.ca (O.Y.); 2 Department of Oncology, Cross Cancer Institute, University of Alberta, Edmonton, AB T6G 1Z2, Canada; john.wilson2@albertahealthservices.ca (J.W.); bjnelson@ualberta.ca (B.J.B.N.); wuest@ualberta.ca (F.W.); 3 Cancer Research Institute of Northern Alberta, University of Alberta, Edmonton, AB T6G 2E1, Canada

**Keywords:** metastatic melanoma, 203/212Pb, melanin, SPECT/CT imaging, radioimmunotherapy, B16F10 melanoma

## Abstract

**Simple Summary:**

Metastatic melanoma is a deadly disease that claims thousands of lives each year despite the introduction of several new drugs into the clinic over the past decade, inspiring the need for novel therapeutics. We investigate targeting melanin pigment, which causes melanoma, with protein molecules called antibodies, which carry a radioactive payload to visualize or treat melanoma tumors. In this study, we imaged and treated melanoma in mice using a c8C3 antibody to melanin and two radioisotopes of lead—Lead-203 for imaging and Lead-212 for therapy. Imaging with Lead-203-bound antibodies allowed for visualization of the tumors in mice, while treatment with Lead-212-bound antibodies slowed down the growth of these aggressive tumors. The treatment was not toxic to mice. We concluded that the melanin-targeting Lead-203/Lead-212-bound c8C3 antibody is a promising agent for imaging and therapy of metastatic melanoma (so-called theranostic), which warrants further investigation.

**Abstract:**

Metastatic melanoma is a deadly disease that claims thousands of lives each year despite the introduction of several immunotherapeutic agents into the clinic over the past decade, inspiring the development of novel therapeutics and the exploration of combination therapies. Our investigations target melanin pigment with melanin-specific radiolabeled antibodies as a strategy to treat metastatic melanoma. In this study, a theranostic approach was applied by first labeling a chimeric antibody targeting melanin, c8C3, with the SPECT radionuclide ^203^Pb for microSPECT/CT imaging of C57Bl6 mice bearing B16-F10 melanoma tumors. Imaging was followed by radioimmunotherapy (RIT), whereby the c8C3 antibody is radiolabeled with a ^212^Pb/^212^Bi “in vivo generator”, which emits cytotoxic alpha particles. Using microSPECT/CT, we collected sequential images of B16-F10 murine tumors to investigate antibody biodistribution. Treatment with the ^212^Pb/^212^Bi-labeled c8C3 antibody demonstrated a dose-response in tumor growth rate in the 5–10 µCi dose range when compared to the untreated and radiolabeled control antibody and a significant prolongation in survival. No hematologic or systemic toxicity of the treatment was observed. However, administration of higher doses resulted in a biphasic tumor dose response, with the efficacy of treatment decreasing when the administered doses exceeded 10 µCi. These results underline the need for more pre-clinical investigation of targeting melanin with ^212^Pb-labeled antibodies before the clinical utility of such an approach can be assessed.

## 1. Introduction

**Figure 1:**
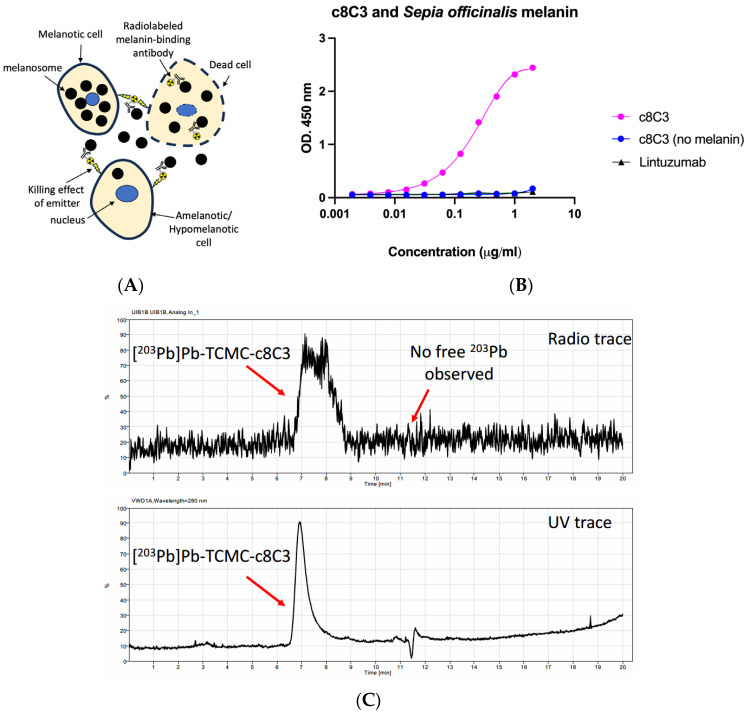
Binding of a melanin-specific antibody to melanin in a melanoma tumor and its quality control: (**A**) A diagram illustrating the process of treating melanoma using a radiolabeled melanin-binding monoclonal antibody is depicted. In a rapidly growing tumor, melanin is released from melanoma cells, which become non-viable as a result of cellular turnover. The melanin-binding antibody selectively binds to the free melanin and delivers cytotoxic radiation to the surrounding area. The radiation emitted in a 360-degree sphere results in the destruction of melanized, weakly melanized, and amelanotic cells through a “cross-fire” effect (adapted from Ref. [14]); (**B**) melanin ELISA showing c8C3 binding to melanin from *Sepia officinalis*. Humanized anti-CD33 antibody lintuzumab was used as a negative control; (**C**) radioHPLC of ^203^Pb-c8C3 shows all radioactivity being associated with the antibody peak.

Metastatic melanoma remains a deadly disease, claiming thousands of lives each year despite several immunotherapeutic agents introduced into the clinic over the past decade [1,2,3,4,5,6,7,8,9,10,11]. This motivates the development of novel approaches and combination therapies for treating metastatic melanoma. One such approach involves targeting melanin pigment with radionuclide therapy using either melanin-binding small molecules or melanin-specific monoclonal antibodies (mAbs) [12]. Our investigations have targeted melanin with melanin-specific radiolabeled antibodies, a method termed radioimmunotherapy (RIT), as a possible strategy to treat metastatic melanoma [12]. In mammalian cells, melanin is located intracellularly inside melanosomes. Figure 1A illustrates the process of treating melanoma using a radiolabeled melanin-binding monoclonal antibody. In a rapidly growing tumor, melanin is released from melanoma cells, which become non-viable as a result of cellular turnover. The melanin-binding antibody selectively binds to the free melanin and delivers cytotoxic radiation to the surrounding area. The radiation emitted in a 360-degree sphere results in the destruction of melanized, weakly melanized, and amelanotic cells through a “cross-fire” effect [13,14]. We previously showed that a human mAb targeting melanin labeled with the short-lived alpha-emitter bismuth-213 (^213^Bi, 46-min half-life) was more efficacious and safer in treating experimental melanoma compared to the same mAb labeled with the long-lived beta-emitter lutetium-177 (^177^Lu, 6.7-day half-life) [15]. This suggests that alpha-emitting isotopes have a strong potential to treat metastatic melanoma.

Alpha-emitters possess high linear energy transfer and a short emission path length of several cell diameters in tissue, which results in significant induction of DNA double-strand breaks and more localized destruction of cancer cells compared to beta-emitters [16,17]. Moreover, there is potential to enhance cancer cell destruction by combining radionuclide therapy with the synthetic lethality characteristics of therapeutic agents and the malfunctioning DNA double-strand break repair mechanisms often found in cancer cells [18]. A short physical half-life of a radionuclide results in a high dose rate, which is necessary to counteract the growth of aggressive cancers during targeted radionuclide therapy. Short-lived alpha-emitters are particularly desirable for the treatment of aggressive cancers, such as metastatic melanoma. Lead-212 (^212^Pb, 10.6-hour half-life) acts as an “in vivo generator” as it decays to alpha-emitters bismuth-212 (^212^Bi, 1-hour half-life) and polonium-212 (^212^Po, 0.3-microsecond half-life), which are both highly cytotoxic [19]. An additional advantage for ^212^Pb is the potential use of lead-203 (^203^Pb, 52-hour half-life) as a SPECT radioisotope, thus making ^203^Pb/^212^Pb a true theranostic pair [19]. Since ^203^Pb and ^212^Pb are chemically identical isotopes, compounds radiolabeled with ^203^Pb/^212^Pb should exhibit similar in vivo biodistributions, increasing confidence that the therapeutic ^212^Pb dose is precisely delivered to tumors delineated in ^203^Pb diagnostic SPECT scans. In the current study, we applied a theranostic approach to microSPECT/CT imaging and RIT of experimental B16-F10 murine melanoma with a ^203^Pb/^212^Pb labeled chimeric antibody targeting melanin.

## 2. Materials and Methods

Antibodies, conjugation, and radiolabeling. Aragen Bioscience manufactured the chimeric antibody (c8C3) that binds melanin. Human IgG control (Cat. # DAGIC1333) was purchased from Creative Diagnostics; humanized anti-CD33 antibody lintuzumab biosimilar—from Creative Biolabs (Shirley, NY, USA). The immunoreactivity of c8C3 mAb towards melanin was measured by in-house melanin ELISA using melanin from *Sepia officinalis* (cat# M2649, Sigma-Aldrich, St. Louis, MO, USA).

The bifunctional chelating agent TCMC (2-(4-isothiocyanatobenzyl-1,4,7,10-tetraaza-1,4,7,10,tetra-(2-carbamonylmethyl)-cyclododecane) was purchased from Macrocyclics (Plano, TX, USA). The ^224^Ra/^212^Pb generator was supplied by Los Alamos National Laboratory (Los Alamos, NM, USA). ^203^Pb was supplied by the Medical Isotope and Cyclotron Facility at the University of Alberta. The ^203^Pb was produced by irradiation of isotopically enriched ^205^Tl metal and the ^205^Tl(p,3n)^203^Pb nuclear reaction on a TR-24 cyclotron and purified to a ^203^Pb(OAc)_2_ chemical form amenable for direct radiolabeling at 22 °C, as previously described [20]. c8C3 and human IgG mAbs were conjugated to *p*-SCN-TCMC as previously described [15], with an initial 20 molar excess of *p*-SCN-TCMC over c8C3 mAb. Chelating agent to antibody ratio (CAR) for the resulting antibody conjugate was determined via the MALDI-TOF method at the University of Alberta, Canada, mass spectrometry facility and found to be ~8.8 TCMC/mAb.

Murine B16-F10 melanoma model. All animal studies were approved by the Animal Research Ethics Board of the University of Saskatchewan, Animal Protocol # 20170006. Six-week-old C57BL/6 female mice obtained from Charles River Laboratories (Wilmington, MA, USA) were injected subcutaneously with 5 × 10^5^ B16-F10 murine melanoma cells in Matrigel (1:1 dilution, Corning Inc., Coring, NY, USA) into the right flank. The microSPECT/CT imaging and RIT studies were performed when tumor volume reached 50–75 mm^3^.

Radiolabeling of TCMC-c8C3. A ^224^Ra/^212^Pb generator was purchased from Los Alamos National Laboratory (Los Alamos, NM, USA). The generator was eluted according to the manufacturer’s instructions. In summary, 1 mL of 0.5 M HCl was first passed through the column to elute any ^212^Bi, followed by 1 mL of H_2_O to wash the column. Immediately after the water wash, 1 mL of 2 M HCl was passed through the column to elute ^212^Pb. Finally, 1 mL of H_2_O was passed through the column to wash the generator of any remaining HCl. 0.5 mL of H_2_O was added to the generator for storage for subsequent use, leaving the resin immersed in H_2_O. The 2 M HCl ^212^Pb elution was collected in 0.25 mL fractions, with the first fraction being discarded. The remaining fractions were combined and evaporated at 85 °C under a flow of nitrogen gas for 1 h. The dried fraction was redissolved in 0.15 M NH_4_OAc buffer (pH = 6.5), and the activity of ^212^Pb was calculated using a CRC-25W (Capintec, Inc., Florham Park, NJ, USA) using a provided multiplication factor with t = 0 set as the elution time. The desired activity of ^212^Pb was added to the TCMC-c8C3 to achieve a 5:1 µCi:µg specific activity and reacted at 37 °C for 1 h. Radiochemical yields were found to be greater than 98% via iTLC (Agilent, Santa Clara, CA, USA), and the radiolabeled product was used without further purification. A ^203^Pb solution in 1 M NH_4_OAc buffer was received from the University of Alberta (Edmonton, AB, Canada) and used directly to achieve a 5:1 µCi:µg specific activity with TCMC-c8C3 dissolved in 0.15M NH_4_OAc buffer, with radiolabeling performed at 37 °C. Radiochemical yields were greater than 99% as confirmed by iTLC and SEC-HPLC (Agilent, Santa Clara, CA, USA) equipped with a TSKgel SuperSW2000xl, 4.6 mm ID x 30 cm, 4 µm column (Tosoh Bioscience, Tokyo, Japan), running an isocratic mobile phase of 50 mM sodium phosphate buffer and 200 mM NaCl at pH = 7.0.

microSPECT/CT imaging of B16-F10 melanoma with ^203^Pb-c8C3 mAb. B16-F10 tumor-bearing mice were injected via the tail vein with 200 µCi ^203^Pb-c8C3 (syringes were measured before and after injection to account for a total administrated dose that ranged from 204 to 190 µCi) and imaged at 3, 24, 48, and 120 h on a MiLabs VECTor^4^ (Utrecht, The Netherlands) using an Extra Ultra High Sensitivity Mouse (XUHS-M) collimator for 20–350 keV. Images were processed using MiLabs software (v8.00RC6) using a 0.4 mm voxel grid with 10 iterations and 10 subsets. SPECT images were then filtered using Gaussian smooth 3D FWHM (2 mm in X, 2 mm in Y, and 2 mm in Z) using pMOD v3.903 (pMOD Technologies, Inc., Zurich, Switzerland). Tumor regions of interest (ROIs) were drawn based on CT images using 3D Slicer v5.0.3 (slicer.org). Images were exported as RTSS dicom files and imported into pMOD for SUV analysis. SUVbw was calculated in pMOD, with SUVbw = r/(a’/w), where r is the activity concentration in the ROI (kBq/mL), a’ is the decay-corrected dose of ^203^Pb-c8C3 (kBq), and w is the body weight (kg) of the mouse. Images were then generated with an SUV range of 1–2.5 g/mL for direct comparison between time points.

RIT of B16-F10 melanoma with ^212^Pb-c8C3 mAb. Tumor-bearing mice were randomized in groups of 5 animals (except for an untreated control group that had 10 animals) and administered via the tail vein with 5, 10, or 17 µCi ^212^Pb-c8C3 mAb, 10 or 17 µCi ^212^Pb-IgG control mAb, or left untreated. Tumor size and animal weight were measured and documented twice per week. Blood was collected from mice at the end of the study for blood cell counting and blood chemistry analysis.

Statistical analysis. The determination of statistical power for in vivo cytotoxicity experiments was computed using PASS version 11 (NCSS, Inc., Kaysville, UT, USA). The estimation was based on pilot data and cautious assumptions concerning the groups treated with radiolabeled antibodies, employing diverse simulations of tumor volumes. The results of all simulations indicated a minimum power of 83%. This outcome, combined with the large differences observed between the treated and untreated animals, enabled the use of only five mice per group for the in vivo studies. All data was analyzed using GraphPad Prism (Version 8.3.1). One-way ANOVA was used for the analysis of the tumor volumes, followed by Dunnett’s post hoc analysis to compare all treatment groups vs. the untreated group. Mice survival was analyzed using Log-rank text.

## 3. Results

TCMC-conjugated c8C3 mAb to melanin demonstrated binding to melanin and quantitative radiolabeling with ^203^Pb/^212^Pb. TCMC-conjugated c8C3 mAb preserved its binding to melanin, as shown by melanin-binding ELISA (Figure 1B). RadioHPLC of ^203^Pb-c8C3 revealed that all radioactivity was associated with the antibody peak, thus confirming the quantitative radiolabeling yields determined by iTLC (Figure 1C).

**Figure 2:**
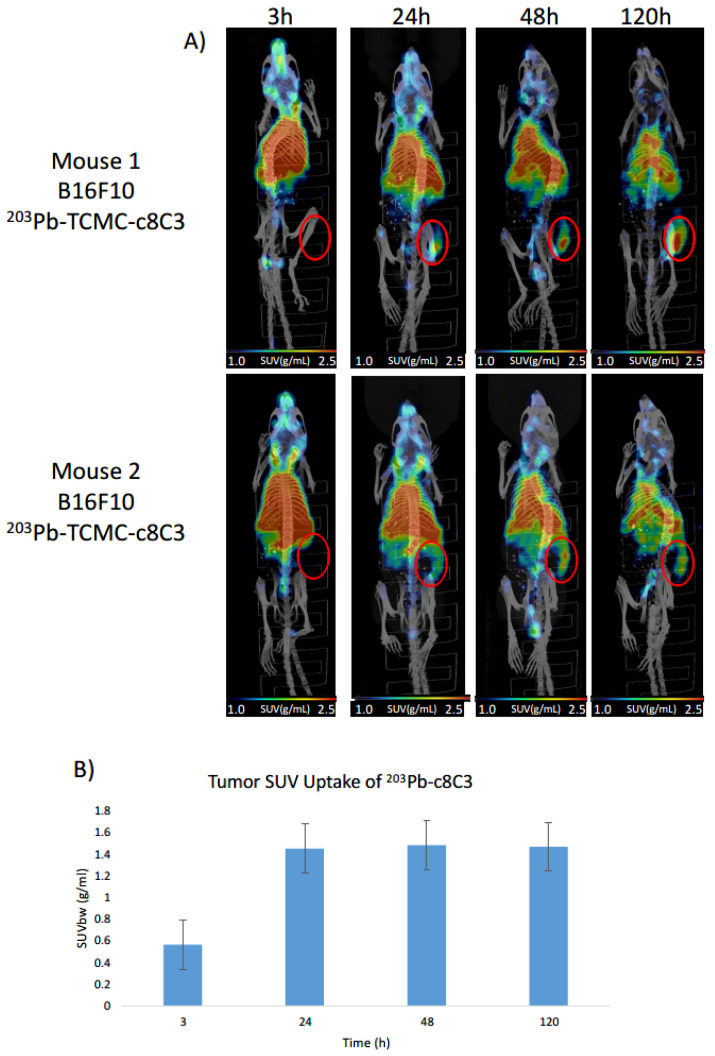
(**A**) microSPECT/CT imaging of B16-F10 melanoma-bearing mice at 3–120 h post-^203^Pb-c8C3 mAb administration. The SUVbw values are standardized with a range of 1–2.5 g/mL, as shown below the images. The tumors are identified with red circles. (**B**) Measured SUVbw values based on Tumor ROIs are drawn using CT Data.

^203^Pb-c8C3 demonstrated high localization in B16-F10 melanoma tumors by microSPECT/CT. Figure 2 displays the microSPECT/CT images of B16-F10 tumor-bearing mice at 3, 24, 48, and 120 h post-mAb administration (Figure 2A) as well as tumor SUV values (Figure 2B). The ^203^Pb-c8C3 stayed in circulation for up to 120 h post-injection (p.i.). The tumor uptake of ^203^Pb-c8C3 increased from 3 to 24 h, after which the uptake remained mostly constant up to 120 h p.i. with little washout of radioactivity.

**Figure 3:**
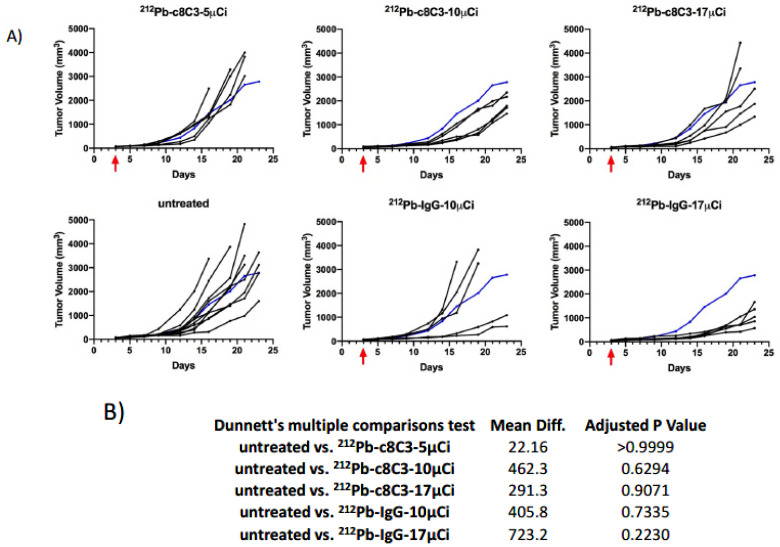
Tumor volume of B16-F10 tumor-bearing mice after RIT. The black line represents the individual tumor volume of the mouse. The blue line represents the average untreated tumor volume. (**A**) A single dosage (5 μCi, 10 μCi, or 17 μCi) of ^212^Pb-labeled c8C3 anti-melanin antibody or IgG control antibody was given to the treatment groups. The red arrow indicates the time of RIT. (**B**) *p* values for comparison of tumor volumes to the untreated group.

**Figure 4:**
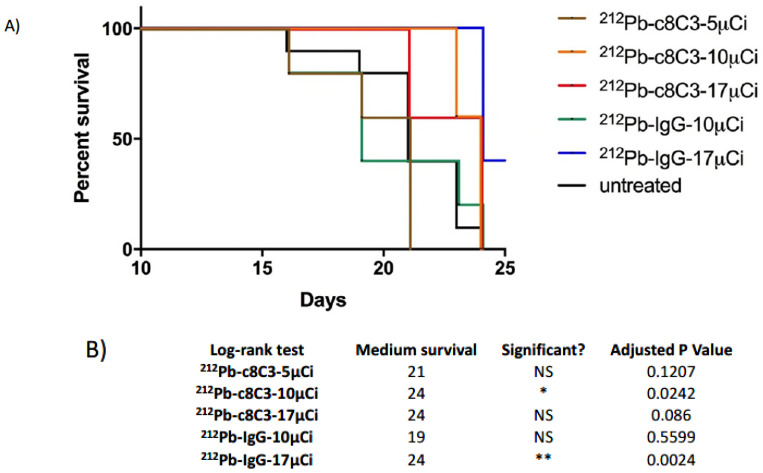
Kaplan-Meier survival curves for B16-F10 melanoma-bearing mice treated with the melanin-targeting ^212^Pb-c8C3 mAb. (**A**) survival curves; (**B**) *p* values for comparison of survival in treated groups relative to the untreated group. * indicates a statistically significant p value smaller than 0.05, ** indicates a statistically significant p value smaller than 0.005.

^212^Pb-c8C3 mAb slowed B16-F10 tumor growth in a dose-dependent manner. Figure 3A depicts the individual tumor volume in mice with B16-F10 melanoma tumors after treatment with a single dose of ^212^Pb-c8C3 or ^212^Pb-IgG control (red arrow) three days after tumor cell inoculation. The majority of untreated mice reached the maximum tumor size or became ulcerated within 20 days of tumor induction. All mouse-bearing tumors reached the study-defined endpoint within 24 days. The lower dosage (5 μCi) of ^212^Pb-c8C3 treatments did not affect tumor growth when compared to the untreated mice (the blue line represents the average tumor volume of the untreated group). Higher dosages (10 μCi or 17 μCi) of ^212^Pb-c8C3 treatments suppressed tumor growth for up to 18 days. For the non-binding IgG control groups, mice that received a 10 μCi dose showed similar results to the untreated group’s tumor growth, while the group that received the 17 μCi dose displayed a suppression effect on tumor growth. Despite large differences in mean tumor volume between untreated controls and the groups treated with higher doses of RIT, statistical significance was not reached (Figure 3B). In terms of overall survival, 100% of mice in the 10 μCi ^212^Pb-c8C3 and the 17 μCi ^212^Pb-IgG groups survived for 23 and 24 days, respectively, while the other groups displayed significantly shorter survival times (Figure 4A), with longer survival in the former two groups than in the untreated controls (Figure 4B).

**Figure 5:**
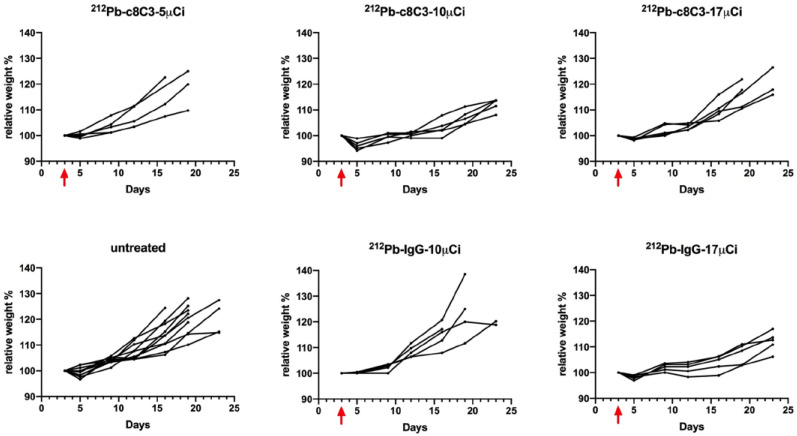
Relative body weight of the B16-F10 tumor-bearing mice after RIT with the ^212^Pb-c8C3 melanin-targeting mAb. The relative weight was calculated based on the starting date of the experiment. Mice in all groups gained weight after the radiation therapy. Red arrow indicates the day of ^212^Pb injection.

Treatment with the ^212^Pb-c8C3 mAb was well tolerated. All mice experienced a 2–8% drop in body weight post-RIT, with the nadir occurring around day 3 post-treatment (Figure 5). Body weight recovery was observed after this point for all groups of mice that received RIT, including those that received the highest dose of 17 μCi. No differences were observed between the groups (Figure 5).

**Figure 6:**
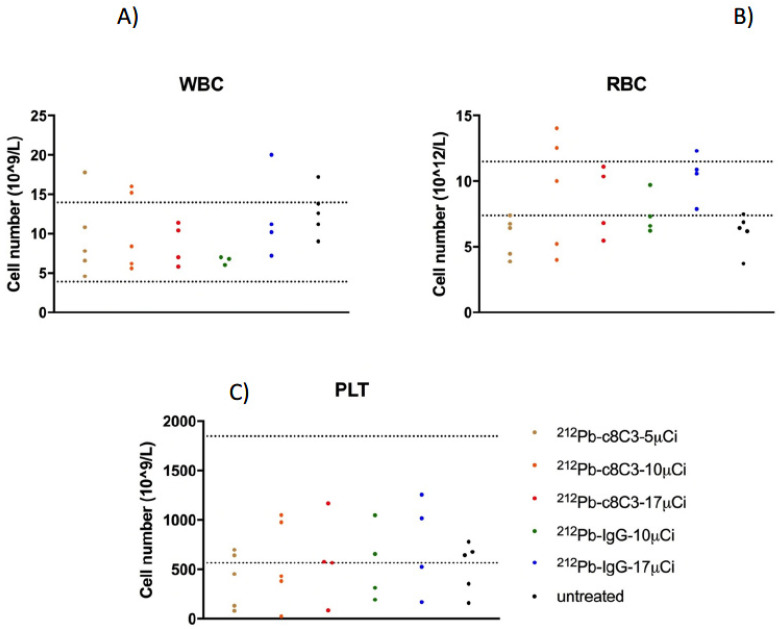
Blood counts in B16-F10 melanoma-bearing mice at the conclusion of the RIT experiment. (**A**). White blood cell (WBC) numbers in each group. (**B**). Red blood cell (RBC) numbers in each group. (**C**). Platelets (PLT) number in each group. The dotted lines show the normal range for white blood cells, red blood cells, and platelets in female C57Bl6 mice.

**Figure 7:**
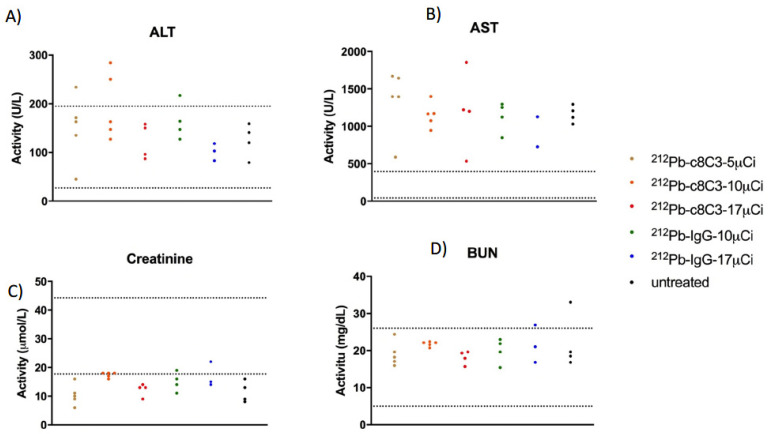
Liver and kidney functions were tested in B16-F10 melanoma-bearing mice at the conclusion of the RIT experiment. (**A**,**B**), liver enzymes (aspartate transaminase (AST) and alanine transaminase (ALT)) activity were tested. Elevated enzyme activity would indicate liver damage. (**C**,**D**). creatinine, and blood urea nitrogen (BUN) level were tested. Increased level of Creatinine or BUN would indicate kidney damage or dysfunction. The dotted lines show the normal range for AST, ALT, creatinine, and BUN for female C57Bl6 mice.

At the completion of the study, a complete blood count (CBC) and blood chemistry analysis were performed for each mouse. The white blood cell (WBC), red blood cell (RBC), and platelet (PLT) values were compared between treatment groups and the untreated control (Figure 6). The observed CBC values were within the normal range for each cell type, with no differences between the treated groups and untreated controls, suggesting that the hematopoietic system was able to recover from the radiation therapy. Aspartate transaminase (AST) and alanine transaminase (ALT) levels were within the range of untreated groups, indicating no detrimental impact on liver function (Figure 7A,B). Similarly, creatinine and BUN values were unaffected by RIT (Figure 7C,D), indicating that kidney function was uncompromised. No significant differences were observed in CBC (Figure 6) and blood chemistry analysis (Figure 7) between untreated controls and treated groups, suggesting an absence of systemic toxicity associated with melanin targeting ^212^Pb-c8C3 RIT.

## 4. Discussion

In this study, we investigated the use of the ^203^Pb/^212^Pb theranostic pair labeled with the chimeric c8C3 anti-melanin mAb for microSPECT/CT imaging and RIT in B16-F10 murine melanoma-bearing mice. The microSPECT/CT imaging with ^203^Pb-c8C3 revealed a long circulation time of the mAb in the blood, which extended up to 120 h post-mAb administration (Figure 2B). Due to this extended circulation, we observed that maximal uptake in the tumor was achieved after 24 h, and there was no appreciable washout of ^203^Pb-c8C3 from the tumor at later time points, indicating stable uptake. We did not employ an irrelevant radiolabeled antibody control in our imaging experiments since, as we pointed out in our prior work on targeting other intracellular antigens with the radiolabeled antibodies, any radiolabeled antibody targeting an intracellular antigen would exhibit therapeutic effects to some degree, and antibodies targeting surface antigens demonstrate entirely distinct (accelerated) binding kinetics in vivo [21]. High tumor uptake of ^212^Pb-c8C3 would be beneficial for the delivery of tumoricidal alpha radiation to the tumor cells. Despite the long circulation of the ^203^Pb/^212^Pb-c8C3 mAb in the blood, there were no indications of toxicity to the bone marrow, as reflected in the normal values for all CBC parameters (Figure 5), or to the liver and kidneys, as indicated by nominal blood chemistry analysis (Figure 7).

The doses of ^212^Pb-c8C3 used in our RIT experiments were comparable to doses of ^212^Pb-labeled antibodies used by other groups in pre-clinical studies [22,23]. The RIT results of B16-F10 melanoma tumors revealed a dose-dependent pattern where a 5 µCi dose of ^212^Pb-c8C3 resulted in no meaningful effect on tumor growth, while a 10 µCi dose slowed tumor growth compared to either the 10 µCi dose of non-specific control human IgG or the untreated control group (Figure 3). The 10 µCi dose also significantly prolonged the survival of treated mice (Figure 4). Interestingly, when the dose was increased to 17 µCi of either ^212^Pb-c8C3 or ^212^Pb-IgG, the efficacy of ^212^Pb-c8C3 decreased while the efficacy of ^212^Pb-IgG increased. The radiobiology of targeting internal or highly shed antigens with radioimmunotherapy is significantly more complex than targeting surface antigens [24], which leads to different, non-linear pharmacokinetics of tumor uptake and, as a result, biphasic therapy efficacy, as observed in this study. In this regard, when surface antigens are targeted with a radiolabeled antibody, killing of those cells leads to target depletion, which makes it easier for incoming radiolabeled molecules to penetrate deeper inside the tumor to deliver their cytocidal payload. However, in the case of internal antigens such as melanin, which is reachable for antibody molecules only in leaky and/or already dead cells, the killing of cells with radioimmunotherapy creates more and more antigen, which will eventually lead to the formation of an “antigen barrier”, thus preventing the radiolabeled antibody molecules from penetrating deeper inside the tumor [24]. We have demonstrated using computer modeling that because of the “antigen barrier”, the radiation doses delivered to the tumors by the beta-emitter-labeled antibody to melanin are practically the same for the tumors with drastically (100-fold) different melanin contents [25]. The “antigen-barrier” effect influences the outcomes of therapy even more significantly when alpha-emitting radionuclides with their short range in tissue, such as ^212^Pb/^212^Bi, are used, as alpha-particles cannot penetrate deep into the tumor if emitted in the vicinity of the antigen-barrier. This results in biphasic therapy efficacy, as we observed with the 17 µCi dose of ^212^Pb/^212^Bi-c8C3. In contrast, the non-binding IgG control freely penetrates deep into the tumor due to the EPR (enhanced permeability and retention) effect [26], thereby delivering alpha radiation randomly to cancer cells. The similar effects of specific and non-specific antibodies on tumors when radiolabeled with powerful short-lived alpha-emitters have been reported [27], and the authors explained their observations by variations among animal models, the innate radiosensitivities of tumors, and the need to evaluate radioimmunoconjugates across multiple models. In general, designing meaningful controls for pre-clinical radioimmunotherapy studies is more challenging than for traditional antibody-drug conjugates (ADC), as the latter do not deal with the off-target cytocidal effects of ionizing radiation [28,29,30]. Taken together, these results underline the importance of careful dosing of radioimmunotherapeutic agents and highlight the reality that the highest tolerated doses might not always be the most effective ones.

The ^203^Pb/^212^Pb theranostic pair has shown promise in the treatment of experimental melanoma and prostate cancer in conjunction with short peptides or small molecules [31,32,33]. In the Li et al. metastatic malignant melanoma study, the combination of a ^212^Pb conjugated melanocortin-1-receptor (MC1R) peptide ligand, a BRAF inhibitor (direct inhibition of BRAF protein), and a histone deacetylase inhibitor resulted in increased levels of MC1R, thus increasing MC1R-MC1L binding, which in turn enhanced ^212^Pb uptake in human melanoma cells in the mouse model, decreased tumor growth, and ultimately increased survival [34]. Miao et al. utilized ^203^Pb-labeled alpha-melanocyte-stimulating hormone peptides as an imaging probe for melanoma detection [35,36]. When treating B16-F1 melanoma-bearing mice with a ^212^Pb-labeled peptide, they observed a dose-dependent increase in mouse survival, with 45% of mice becoming disease-free after 200 µCi ^212^Pb[DOTA]-Re(Arg(11))CCMSH [37]. In a different approach to ^212^Pb delivery to melanoma tumors, Pikul et al. showed increased killing of B16-F10 melanoma cells with intracellularly delivered liposomes containing both ^212^Pb and ^212^Bi [38]. We are not aware of any prior mAb radiolabeled with ^203^Pb/^212^Pb that is used experimentally or clinically for the therapy of metastatic melanoma. However, valuable clinical information on the safety of ^212^Pb-labeled antibodies comes from the studies by Meredith et al., who performed a dose escalation clinical trial (five doses, between 7.4 and 21.1 MBq/m^2^ inclusive) and confirmed the safety of trastuzumab conjugated to ^212^Pb when treating peritoneal carcinomatosis [39]. The same group also showed that in a Phase 1 trial of HER2+ ovarian cancer targeted by trastuzumab conjugated to ^212^Pb, there was a significant accumulation of ^212^Pb in cancer cells [40]. They did not observe any side effects and concluded that alpha-emission produced a superior therapeutic effect at the target site [40]. These safety data will help to determine the dose range for future studies of ^212^Pb-mAbs in melanoma patients.

## 5. Conclusions

MicroSPECT/CT with the ^203^Pb-c8C3 mAb enabled imaging of B16-F10 murine melanoma tumors, and the ^212^Pb/^212^Bi-labeled c8C3 antibody in the 5–10 µCi dose range demonstrated a dose-response in slowing down the tumor growth rate and increasing the survival when compared to the untreated and radiolabeled control antibody groups. The treatment with ^212^Pb-c8C3 was also well tolerated, with no observed hematologic or systemic toxicity associated with treatment. However, the work also revealed the complexities of targeting internal antigens such as melanin with a short-range alpha-emitting radionuclide, which resulted in a biphasic tumor dose response with the efficacy of treatment decreasing when the administered doses exceeded 10 µCi. These results underline the need for more pre-clinical investigation of targeting melanin with ^212^Pb-labeled antibodies before the clinical utility of such an approach can be assessed.

## Data Availability

The data presented in this study are available in this article.
